# IL-17A rs1974226 GG genotype is associated with increased susceptibility to Gram-positive infection and mortality of severe sepsis

**DOI:** 10.1186/cc10609

**Published:** 2012-03-20

**Authors:** T Nakada, J Russell, J Boyd, K Walley

**Affiliations:** 1 University of British Columbia, Vancouver, Canada

## Introduction

IL-17A plays a key role in host defense against microbial infection including Gram-positive bacteria. Genetic factors contribute to the host defense. Whether genetic variation of IL-17A is associated with altered clinical outcome of severe sepsis is unknown.

## Methods

We tested for genetic association of IL-17A SNPs with susceptibility to infection and clinical outcome of severe sepsis using two cohorts of European ancestry (St Paul's Hospital (SPH) derivation cohort, *n *= 679; Vasopressin and Septic Shock Trial (VASST) validation cohort *n *= 517). The primary outcome variable was susceptibility to Gram-positive bacterial infection. The secondary outcome variable was 28-day mortality.

## Results

Of four tested tag SNPs (rs4711998, rs8193036, rs2275913, rs1974226) in the IL-17A gene, rs1974226 SNP was associated with altered susceptibility to Gram-positive bacterial infection in the derivation cohort (corrected *P *= 0.014). Patients who have the GG genotype of the rs1974226 SNP were more susceptible to Gram-positive bacterial infection, compared to the AG/AA genotype in the two cohorts of severe sepsis (SPH, *P *= 0.0036; VASST, *P *= 0.011) and in the subgroup having lung infection (*P *= 0.017). Furthermore, the G allele of the IL-17A rs1974226 SNP was associated with increased 28-day mortality in two cohorts (SPH, adjusted OR 1.44, 95% CI 1.04 to 2.02, *P *= 0.029; VASST, adjusted OR 1.67, 95% CI 1.17 to 2.40, *P *= 0.0052).

## Conclusion

IL-17A genetic variation is associated with altered susceptibility to Gram-positive infection and 28-day mortality of severe sepsis.
